# Surgical resection for gastrointestinal stromal tumors (GIST): Experience on 25 patients

**DOI:** 10.1186/1477-7819-6-27

**Published:** 2008-02-27

**Authors:** Luigi Boni, Angelo Benevento, Gianlorenzo Dionigi, Francesca Rovera, Renzo Dionigi

**Affiliations:** 1 Department of Surgery, University of Insubria, Ospedale di Circolo e Fondazione Macchi, Varese, Italy

Our article previously published in *World Journal of Surgical Oncology *[1] was prepared as an update to an earlier article published in *Scandinavian Journal of Surgery *[2], examining data from a further six patients in addition to the 19 studied in the first article. As such the *WJSO *article was produced in the belief that this information would be of interest to those in the field.

Despite substantial overlap with the article published in *Scandinavian Journal of Surgery*, the original article was not referenced in our *WJSO *article; we would like to apologise to the Editors and readers of *WJSO *for any ambiguity this may have caused.

We would also like to apologise to Dr Besana-Ciani, first author of the original article, as her contributions to the study were not acknowledged in the second article.
